# Mutational scanning of spike RBD protein for enhanced ACE2 affinity emerging Southeast Asia in the late transmission phase

**DOI:** 10.1038/s41598-022-09999-9

**Published:** 2022-04-07

**Authors:** Kanchanok Kodchakorn, Tawan Chokepaichitkool, Prachya Kongtawelert

**Affiliations:** Department of Biochemistry, Faculty of Medicine, Thailand Excellence Center for Tissue Engineering and Stem Cells, Chiang Mai University, Chiang Mai, 50200 Thailand

**Keywords:** Computational biology and bioinformatics, Biological models, Computational chemistry

## Abstract

The COVID-19 pandemic has changed the quality of life and economic systems all over the world, as the virus can be transmitted from human to human via air-droplets. Since the SARS-CoV-2 virus was first identified in 2019, the virus has naturally mutated over time. Southeast Asia is one of the areas in the world that has implemented various procedures and measures to slow down the disease outbreaks. The first cluster of COVID-19 was identified from the tourist-travel history, and then the diversity of coronavirus victims has posed a serious issue of human security on a massive scale. To evaluate whether or not naturally occurring mutations have strengthened the infectivity of SARS-CoV-2, we computed in silico the structural dynamics of the RBD-spike protein mutation enhancing ACE2-binding. When considering emerging variations in Southeast Asia, 14 dominant mutations were analyzed by applying the structural and energetic characterization using MD simulations. The ones in the RBD region displayed higher affinity to ACE2 due to the improved interfacial stability of the RBD β-strand surrounding the ACE2 across salt bridge hotspots. The binding hotspots and structurally conserved conformational-epitopes have been identified, which are deleterious for RBD mutation and ACE2 binding. We present an interactive visualization to facilitate the development of effective neutralizing agents for vaccination, prevention and treatment.

## Introduction

As a deeply serious epidemic situation involving coronavirus disease 2019 (COVID-19), severe acute respiratory syndrome coronavirus 2 (SARS-CoV-2) infectivity is a major concern in disease prevention and economic reopening^1^. The rapid spread of coronavirus has put the world on alert and triggered new lockdowns in many countries to date. Since the start of the pandemic, it was clear that the precise identification of host receptors represented a pivotal step in mechanistically explaining COVID-19; however, the concrete determination of SARS-CoV-2 infectivity is extremely laborious due to its continuous evolution with over a thousand strains of whole genome sequences available in public databases^2,3^. Comparing with previous coronaviruses (SARS-CoV), a plethora of different host receptors are described with numerous roles facilitating viral encroachment. This made the researcher question whether augmentative binding partners may play a role in the SARS-CoV-2 protein development^4^.

An intriguing idea to tackle the SARS-CoV-2 infection is to disrupt the virus entrance passing through the host cell, human angiotensin-converting enzyme (ACE2)^4,5^, by blocking the molecular movements by any fundamental steps, especially via the structural basis of the binding interfaces between them^6–9^. An outstanding number of drug repurposing strategies have started to treat the diseases residing in the rapid rate of pandemic diffusion in line with the low-risk safety information of the approved drugs^10,11^. Small synthetic molecules^12–15^ and monoclonal antibodies^16–18^ have been proposed to be able to bind SARS-CoV-2 spike (S) proteins to disrupt the ACE2 binding that would be reported to play an important role in slowing the virus replication^12^ and infection^19,20^. Despite its important relationship, the receptor-binding domain (RBD) on the S protein is highly variable among the respiratory viruses, reflecting the complex selective impression modeling its evolution. Moreover, RBD mutations have already appeared among SARS-CoV-2 pandemic isolates, including some near the ACE2-binding interface. However, this impacts on the receptor recognition and biological activities remain generally uncharacterized. Therefore, comprehensive knowledge of how mutations impact the SARS-CoV-2 S-RBD would aid efforts to understand viral evolution.

**Figure 1:**
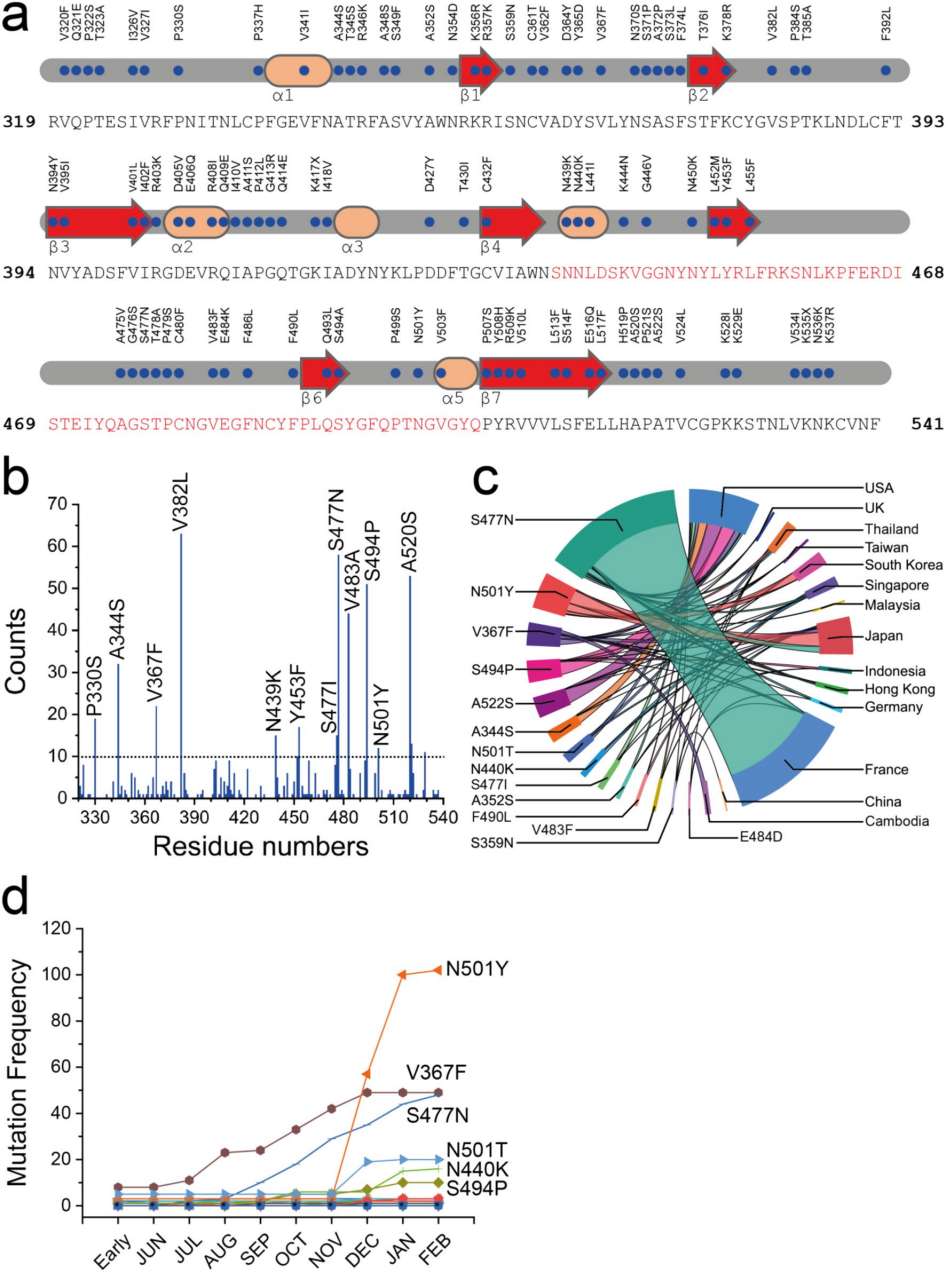
Mutation evolution of SARS-CoV-2 RBD proteins. (**a**) Sequence and secondary structures of SARS-CoV-2 RBD with variants in RBD region. The binding domain sequence is shown in red. (**b**) Distribution of 166 mutant types and nonsynonymous variants in RBD region and their overall mutation counts. All positions containing gaps and missing data were eliminated. (**c**) Chord diagrams representing the correlations between countries and RBD variants, circularly arranged sectors represent RBD mutants and countries. Associations are indicated by intersecting chords. The diagram was created by using Power BI (Microsoft). (**d**) The time evolution of 27 SARS-CoV-2 S protein RBD mutations. Many mutations overlap their trajectories. Here, the collection date of each genome sequence that deposited in GISAID is applied.

Since SARS-CoV-2 was first identified in 2019, thousands of mutations have arisen^21–23^. An availability region of RBD to ACE2 is enforced by the hinge-like configurational change of the S protein^24^. It is of importance to understand SARS-CoV-2 infectivity changes following the existing mutations and to predict the future infection tendencies. Compared with SARS-CoV, the SARS-CoV-2 protein has 725 mutations over its 1255 residues^2,3^, which their sequence identity was found to emphasize only 76%; among them 89 mutations were located on the RBD which has around 194 residues (Fig. 1a). According to the limitations of a comprehensive effect map of SARS-CoV-2 mutations, bioinformatics and molecular modelling on the computational saturation mutagenesis provides a fast systematic procedure to investigate all possible mutation effects on protein structure and function. To address this question of whether these are mutants and why are they causing concern, we analyzed their structural insights into the relative dynamic interactions and stability, and how existing mutations on the RBD protein respect to the potential target sites that displayed emerging infectious disease outbreaks in Southeast Asia from multiple locations including Asia, the UK, Europe, and North America for further designs of antiviral drugs and vaccines against SARS-CoV-2.

## Results

### Mutant clustering and selections

To evaluate the influences of existing S protein mutations on the binding affinity to ACE2, a total of 23,572 whole-genome sequences of SARS-CoV-2 S protein reported by the GISAID database during the late transmission phase from June 2020 to December 2020 were collected^25^. Among them, 357 strains containing variants in RBD region were identified compared to the wild-type Wuhan-Hu-1 strain (GenBank: QHD43415.1)^3^. Following the removal of incomplete, redundant, and ambiguous sequences, all RBD mutants were clustered into 166 mutant types (Fig. 1a). Interestingly, 55 out of 166 mutations are on the RBD region that is in direct contact with ACE2. In deep selections located on the RBD region, the scanning maps of SARS-CoV-2 RBD mutations were analyzed. Total mutations and nonsynonymous variants in RBD are shown in Fig. 1b. These strains were reported from multiple locations including Asia, the UK, Europe, and North America. By pointing to the emergence in ASEAN, 14 RBD mutant types that were circulating in Asia, Europe, and North America were selected (Fig. 1c). In addition, we hypothesize that natural selection favours those mutations that enhance the viral transmission, and if our predictions are correct, the predicted infectivity-strengthening mutations will outpace predicted infectivity-weakening mutations over time.

Figure 1d illustrates the accumulation of the SARS-CoV-2 S-RBD mutations. In the first 3 months of the late transmission phase, only a few infectivity-strengthening mutations were detected. Later on, a few infectivity-weakening mutations gradually appeared, while more infectivity-strengthening mutations occurred. It is interesting to note that overall RBD mutants grew faster, revealing SARS-CoV-2 subtypes having infectivity-strengthening mutations which are able to infect more people in this time period. For Southeast Asian SARS-CoV-2 tracking, six of these were dominant mutant types that were found in more than ten isolates, specifically V367F, N440K, S477N, S494P, N501T, and N501Y, it is apparent that these mutations might have a stronger transmission capacity. This evidence shows that the infection can cause the virus to spread by multiple strategies, such as from a conference held by a company and/or the tour groups from other countries to ASEAN countries in a matter of months, since the outbreak has raised serious concerns about how the region deals with pandemics.

### Mutational scanning and energetic affinity of the binding interfaces with ACE2

**Figure 2:**
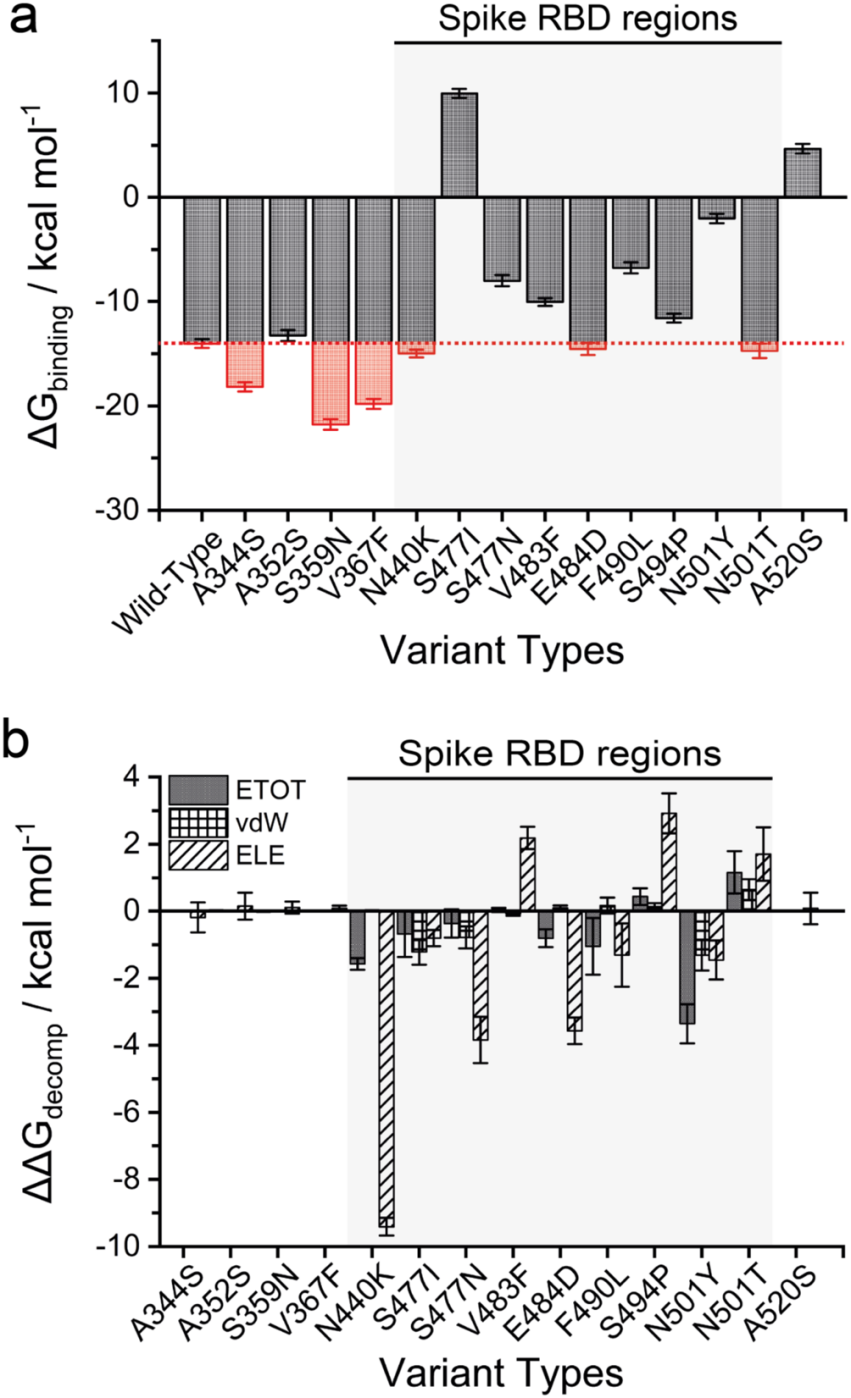
Binding free energies of the variant complexes estimated using MM-PBSA method. (**a**) Binding free energy (ΔG_binding_, kcal mol^−1^) for the interaction between SARS-CoV-2 RBD variants and ACE2 protein. The ΔG calculated for the wild-type of ACE2-spike RBD protein complex are marked in dotted line. Red bars indicate a negative ΔG value related to stronger affinity for the RBD variant binding to ACE2 than that of the wild-type model. (**b**) Per residue free energy decomposition changes (ΔΔG_decomp_, kcal mol^−1^) on the key residues in RBD mutants for the energy components in term of the total binding free energy (ETOT), van der Waals energy (vdW), and the sum of the electrostatic (ELE) interactions of the solvation free energy. Data are presented as mean ± SD. The *p*-values were calculated using single single-tailed student *t*-test. The *p*-values are shown for those with *p* < 0.05.

To clarify the RBD-S mutagenic protein that affects binding to ACE2, we constructed the RBD mutants and scanned the interfacial residues of the binding. Figure 2a shows the binding free energy (ΔG_binding_) of the RBD-S mutants using the molecular mechanics calculation through the MM-PBSA method. Comparing with the wild-type (ΔG_binding_ =  − 14.02 ± 0.401 kcal mol^–1^), six RBD mutants showed a significantly stronger affinity to ACE2. Notably, three of them (A344S, S359N, and V367F) are closely located adjacent to the α/β chains of the RBD region (Fig. 1a) which might be changed the binding interface configuration supporting the higher affinity to ACE2. Besides, three S-RBD mutants (N440K, E484D, and N501T) also showed a binding affinity with lower free energy values in comparison to the wild-type, which is supported by an electrostatically charged side chain of amino acid residue for ACE2 binding^26^. More importantly, the binding stability during MD simulations can be better encouraged by the fluctuation of the structurally individual complex using root mean square deviations (RMSD) with respect to their optimized initial structure (Supplementary Fig. S1). Steady oscillation and small fluctuation of RMSD were observed in the RBD mutant model related to the steady binding free energy, indicating that these mutant complexes were more stable and endured lesser conformational changes during simulations.

Furthermore, we determined the decomposition of free energy on a per residue basis according to the specific contact upon the binding interface identified by the sum of energetic components in terms of the total binding free energy (ETOT), van der Waals energy (vdW), and the sum of the electrostatic (ELE) interactions (Fig. 2b). The negative and positive values of decomposed free energy indicated the favourable and unfavourable contributions for binding, respectively. The potential binding energy of both RBD and ACE2 interface residues will result in the strong interaction in association with the complexes. Upon mutagenesis in the RBD region of S protein, the high affinity caused by interfacial residues exhibited a general decreased free energy decomposition in all energy components, especially in terms of ETOT and ELE through an entropic effect by the disruption of highly dynamic hydrogen bonds between hydrophobic amino acid in RBD β-strand and the helix motif interface of ACE2. The N501Y position contributed the most to the decomposed energy components followed by the N440 and F490 positions in comparison to the wild-type complex. This conserved binding position also corresponds to the regulatory sites in co-evolutionary residue networks that may control signal propagation across binding interfaces. This is to say that the functional relevance of the RBD mutations may be inferred. On the other hand, other mutant positions apart from the RBD region showed slight shifts in the decomposition energy upon the binding of ACE2. This implies that RBD mutations have more potential favourable hotspots involved in the interface interaction.

Based on the per-residue scanning map for each complex, it is indicated that the essential amino acids in RBD interfaces exhibited negative energy influences for ACE2 binding (Supplementary Figs. S2–S5). The potential residues in the RBD region (Y449, S477, Y489, F490, T500 and N501) were verified to have significant effective contributions, with relative energy below 1.5 kcal mol^−1^ related to the wild-type model, for the stabilization energy of the molecular complex. There was strong interdependence of the effects of the individual residues in the RBD sequences. The difference between high and low affinity binding depends on the interaction of ACE2 interfacial residues with the amino acids in the RBD region of the SARS-CoV-2 protein.

### Conformational dynamics for the high affinity mutant model

**Figure 3:**
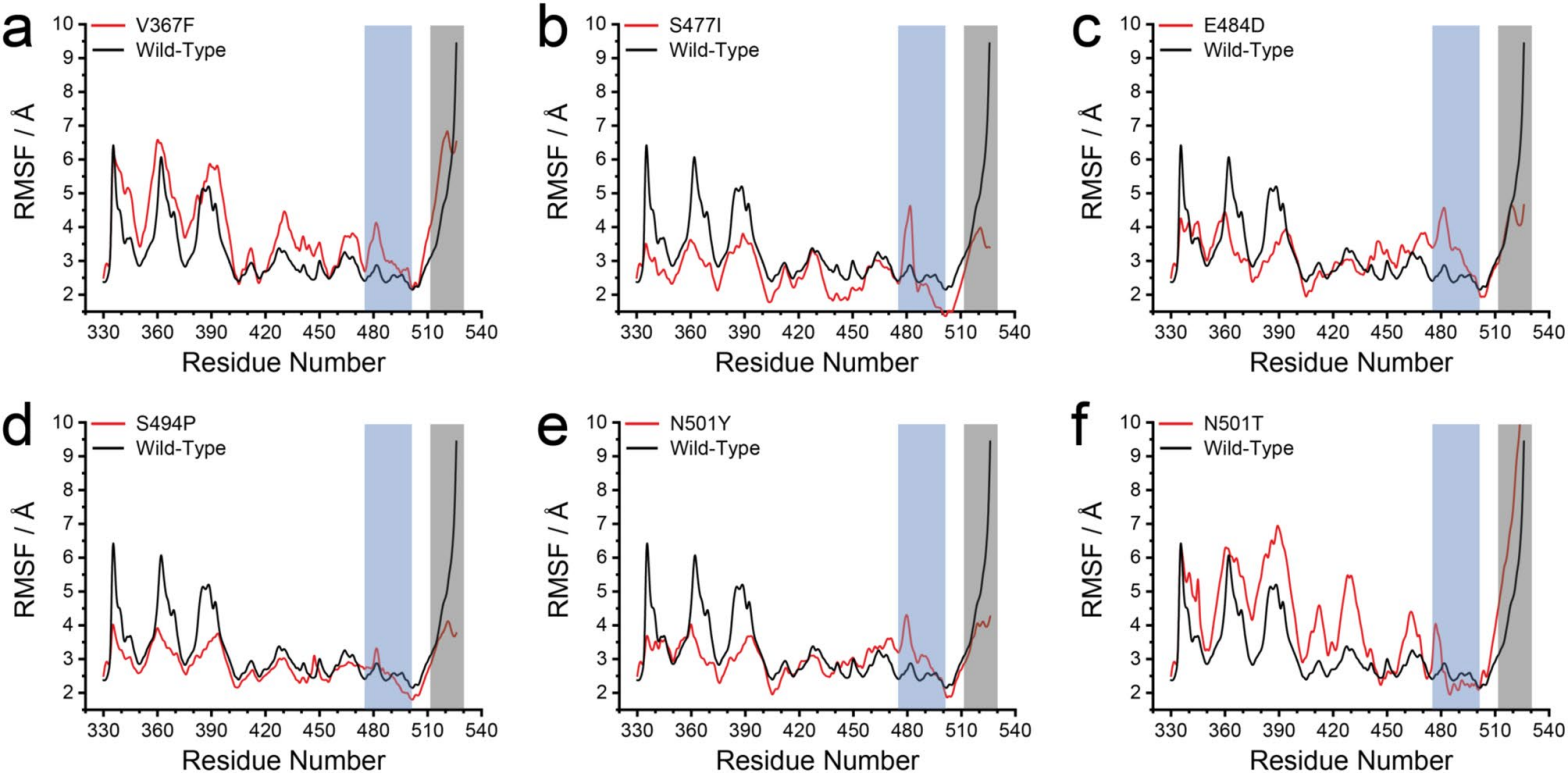
Structural dynamics of RBD mutations on the binding affinity. The root-mean-square fluctuations (RMSF) of spike RBD-ACE2 binding complex; (**a**) V367F, (**b**) S477I, (**c**) E484D, (**d**) S494P, (**e**) N501Y, and (**f**) N501T, in comparison to the wild-type. The RBD interface residues are highlighted in cyan (residues 475–500) and gray (residues 515–525) colors.

To explore the structural analysis of the potential model in sufficient detail, we calculated the dynamics of the residues in terms of the analysis of root mean square fluctuations (RMSF), which is useful to situate the flexible and disordered region as well as the heterogeneity of a system (Fig. 3)^27,28^. As the binding interface residues array in a random coil conformation (Fig. 1a) that lacks structural rigidity, a structural arrangement in this region should be necessary to sustain their configurations of the binding surface, which may facilitate the binding affinity. The residues 465–488 (a random coil near the binding interface), showed a remarkable decrease in RMSF for the V367F, S477I, E484D, S494P, N501Y, and N501T mutant models, indicating that these strong affinity positions stabilized the complex as a whole, resulting in a reduced fluctuation in these interfacial residues surrounding the ACE2 binding residues.

The β-strand conformation (residues 510–524) displayed greater structural rigidity with fewer or similar fluctuations than the wild-type, whereby the substitutions that account for the increasing binding affinity in each mutant are all located near this fragment region. Moreover, the similar affinity mutants, in contrast to the strong binding affinity mutants, also exhibited a structural rigidity of variant complexes in the binding site (Supplementary Fig. S6). It can be noted that the strong binding affinity in RBD mutants does not only depend on the interfacial residue near the active surface but also provides greater stability of individual residues surrounding the potential interface hotspots for conformational searching to fit on to the binding site.

## Discussion

The recent literature reviewed the large number of SARS-CoV-2 mutations that are among the most non-conservative in its genome, in which many of them are located on the RBD region^2^. The sequence-phenotype maps reveal tremendous heterogeneity in mutational constraint across the RBD. Here, the computational approach was used to apply a fast systematic procedure to investigate all possible mutations and identify the potential functional sites related to the virus infectious. Many sites are highly tolerant of mutations with respect to ACE2 binding as well as their expression^22^, whereas other sites are constrained to the wild-type residue. A substantial number of RBD position (residues 344–364) are tolerant of mutations with respect to ACE2 binding which are consistent with folding and stability being global constraints common to many sites.

Mutations that enhance affinity are notable at RBD residues, although these residues are involved in a dense network of polar contacts with ACE2. Our calculations show there is substantial plasticity in this network as mutations that reduce the polar manner of these residues can enhance binding affinity. According to the Fig. 2, two patterns of binding free energies were observed and separated in (i) high ΔG_binding_ form (≥ 10 kcal mol^−1^), and (ii) low ΔG_binding_ form (< 10 kcal mol^−1^). However, our data also indicate that global RBD stability contributes to ACE2-binding affinity. First, the RBD mutant with high binding affinity (K440, F483, D484, P494, and Y501) that the forms that bind the ACE2 receptor quite well, thereby favouring the bound-state due to masking of the host receptor binding site. On the other hand, the low potential variants (N477, L490) destabilize the infectious form of the viral spike protein, resulting in the unliganded form, “down” SARS-CoV-2 viral forms, that would be expected to infect less readily. In general, mutation effects on RBD binding and expression are correlated with residues that deviated from this trend clustering at the ACE2 interface^22^. This correlation between expression and binding is consistent with studies on antibodies where mutations that improve stability and rigidity accompany increases in binding affinity. That is to say, the mechanism by the unliganded-states causes greater mortality may be immunological rather than virological, in other words, this form that binds the receptor less well acts as the better shielded from host immune system attack and/or elicits harmful anti-viral-spike antibodies. Furthermore, these mutation positions were observed in turn-secondary regions, which may result in the structural motif efficiency to bind the host receptor. Outside the RBD region, the mutant was also found to result in substantially high potential affinity scores, which may be disordered in the transition-state conformational changes of the viral spike protein.

**Figure 4:**
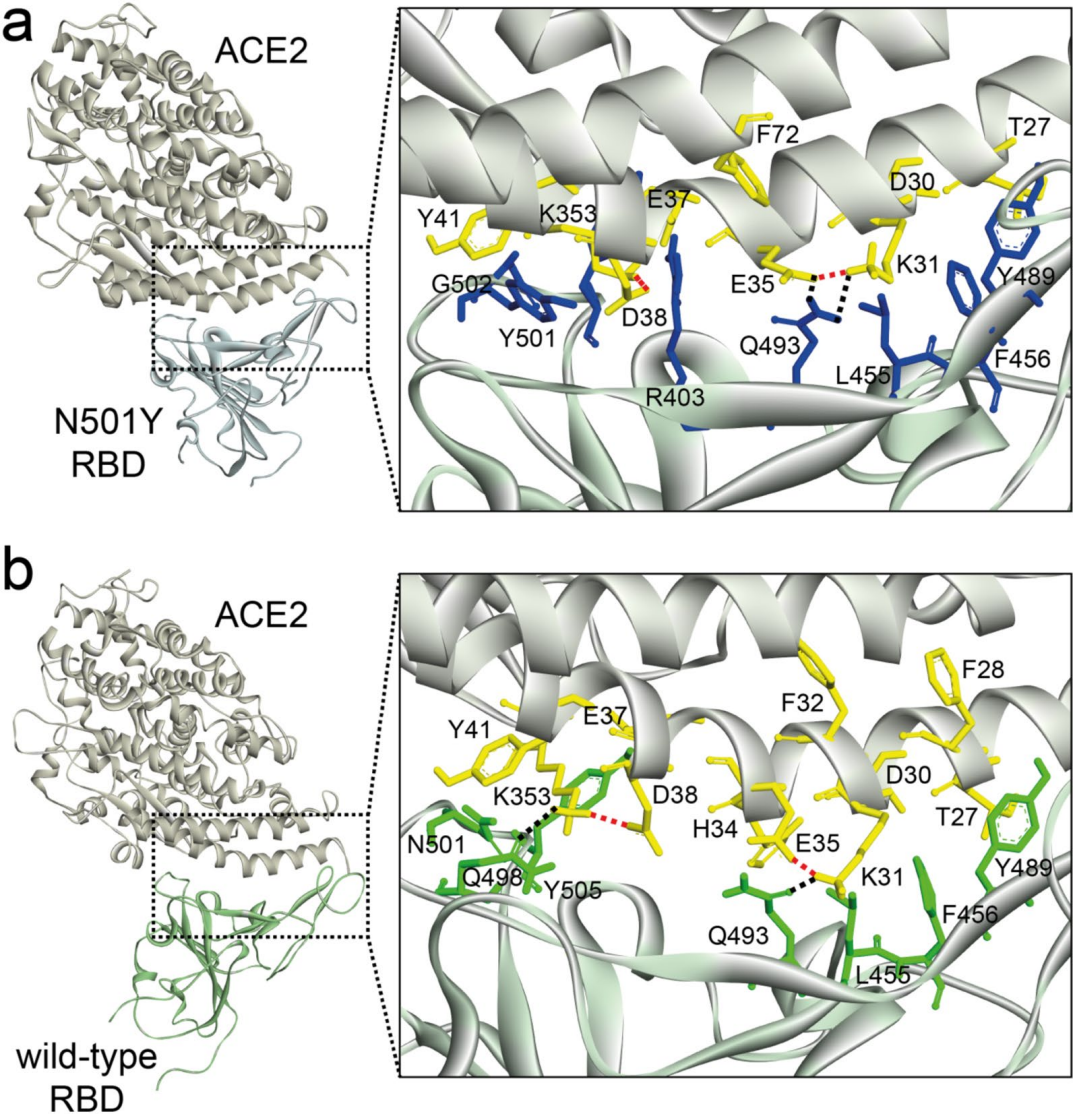
Structural mapping of the binding interface structure of (**a**) N501Y, and (**b**) wild-type complex with ACE2. The structure of RBD is shown in light-yellow ribbon, while ACE2 is in green (wild-type) and pastel-blue (N501Y) ribbon. The close-up of these binding complexes is shown the crucial mutations responsible for high binding interaction (right panel). The interfacial residues of SARS-CoV-2 RBD residues are annotated and shown in green and blue sticks, ACE2 residues are in yellow sticks. The salt bridge presents in red dashed line, while hydrogen bonds are in black dashed line. The figure was drawn by Discovery Studio 2019 Client (Biovia software).

For example (Fig. 4), we provided valuable insights into the interfacial binding mechanism and bound-state characteristics of the SARS-CoV-2 RBD N501Y (UK variant) in comparison with the wild-type complex. Three different regions on RBD protein can be distinguished: (i) the first region (Q498, N501, G502 and Y505) and third region (F456 and Y489) interact with ACE2 α1-helix (C-terminal: T27 and F28, N terminal: Y41, Q42, K353, and R357) chains. The second region of the RBD (R403, L455, Y453, and Q493) binds with the central segment of the α1 helix interface of the ACE2 interface residues (D30, K31, H34, E35 and D38)^9^. The binding complex network of N501Y mutant is vastly different and considerably stronger than the corresponding interface residues following on the wild-type model. The binding network of N501Y mutant interacted to establish more stable via the main interface segment (L455, F456, Y489, F490, and Q493) with the residues of K31 and E35 of ACE2 (Fig. 4a). On the other hand, the stability of salt bridge residue in ACE2 in the wild-type was neutralized by an interfacial residue Q493 (Q493-K31) and Q498 (Q498-K353) of RBD protein, while interaction contacts with hydrophobic residues L455, F456, Y489, and Y505 are more dynamic (Fig. 4b). Indeed, the stability of the hydrogen bridge interacting between Q493 and K31 hotspot was found to conserve as a key amino acid for ACE2 binding^29^. Similarly, mutations to polar and charged amino acids enhance the binding at interface residues A344, S359, N440, and E484, consistent with the destabilizing effect of surface-exposed hydrophobic patches.

An extensive dynamic simulation study revealed a balance of hydrophobic interactions and refined the hydrogen bond networks in SARS-CoV-2 RBD binding interfaces^30^. The RBD consists of a concave surface anchored by a β-hairpin and a disulfide bond stabilizing one of the lateral loops, particularly on RBD residues that cradles the ACE2 α1-helix and a β-hairpin centered on K31 and K353 which are “hotspots” of binding for SARS-CoV-2. The hydrophobic residue of the RBD binding interface can create a salt bridge involving K31 and K353 residues in ACE2 across hydrophobic contact sites and leads to a stronger electrostatic interaction and maintains stability of binding affinity^26,30,31^. Remarkably, the corresponding sites in the N501Y model (Y489 and Q493) are slightly unstable interactions with K31^26^. Although the weak intramolecular salt bridge K31-E35 is broken in the binding interface; the stability and neutralizing capacity of the complex will require maintenance not only by the interaction contacts with hydrophobic residues (L455, F456 and Y489) but also via reinforcement by an extensive network of the other residues surrounding the key hotspots.

The binding free energy values revealed that the interfacial hotspots on ACE2 corresponded to residues of T27, D30, K31, and E35 displaying persistently greater mutation-induced affinity changes in the SARS-CoV-2 RBD mutant complex (Fig. 4a). For this reason, the virus-binding interface hotspots on ACE2 (D30, K31, H34 and E35) may play an important role in anchor docking for SARS-CoV-2 RBD-selective binding. Another hotspot on ACE2 is the K353-D38 salt bridge that is surrounded by hydrophobic walls formed by E37, D38, Y41, and Q42 residues^32^, supporting by R403, Y501, and Y505 in the N501Y model (Fig. 4a) and by Q498, N501, and Y505 (hydrophobic contact) in the wild-type model (Fig. 4b). Although these salt bridges are important for S protein recognition, the decomposed energy contributions of the corresponding residues in the N501Y model showed tightly higher affinity than that of the wild-type model, suggesting that the key hotspots may be more critical for driving the binding selectivity of RBD mutations. Furthermore, the stronger affinity of RBD mutants towards ACE2 caused by the electrostatic contacts interaction can explain the specific phenomenon of SARS-CoV-2 recognition for ACE2 binding. However, there are other surface positions where contribution of RBD stability to ACE2 binding is tolerated or even beneficial for RBD expression in which mutation effects on RBD binding and expression are correlated.

In conclusion, the combination analysis on the structural binding phenomenon and drivers of preferable binding hotspots underlying the affinity mechanism was conducted by leveraging diversity across dynamic simulations that emerged in ASEAN from multiple location databases. By exploring the mode of molecular mechanisms of the viral infection, the structural binding stability and potential binding sites in the middle segment of the helix motif interface can enforce correlated motions in biomolecular couplings for modes of SARS-CoV-2 RBD recognition. The RBD protein is the influential target of neutralizing antibodies to SARS-CoV-2 virus. Although in vitro experiments indicate the RBD is capable of fixing mutations which escape neutralizing antibodies, it is unclear to that extent the RBD will evolve to escape such antibodies of some other viruses^33,34^. Many antibodies have epitopes that overlap the RBD-ACE2 contact interface and are strongly constrained by mutation effects on the binding. A recent study found that the mutations of RBD protein will enable the escape from the binding-directed motifs neutralizing antibodies^33^. Our data indicate that the escape may occur at sites that have high mutational tolerance depending on the RBD binding that motivated the different interaction mechanisms of the SARS-CoV-2 through the RBD’s evolutionary capacity for antibody escape. Despite the fact that the amino acid hotspots in these RBD ACE2-contact interface are constrained for binding stability even in our investigations on the isolated RBD S protein, some of them likely may exhibit augmentative constraint due to the quaternary structure of spike-trimer protein^35,36^. Taken together, our results identify multiple mutationally constrained patches on the RBD surface that may be targeted by antibodies, that could inform the prescription of neutralizing antibody cocktails aiming to limit the emergence of SARS-CoV-2 mutants. Especially, if deep mutational scanning approaches like our study are extended to define more experimental measurements in comparison of a functional and a structural epitope.

## Materials and methods

### Mutation dataset and structure selection

A total information of 23,572 whole-genome sequences of S protein with high coverage of SARS-CoV-2 strains from the infected individuals around the world isolated during the late transmission phase from June 2020 to December 2020 from the GISAID database^25^. The genome sequences with amino acid mutations in RBD region of S protein was parsed and used to analyze in this study and compared to the wild-type Wuhan-Hu-1 strain (GenBank: QHD43415.1)^3^.

Protein structure of RBD region on the S protein and ACE2 were obtained from the RCSB Protein Data Bank: SARS-CoV-2 RBD with ACE2 (PDB ID: 7KMB)^37^. The RBD-ACE2 complex was used to investigate the binding affinity and protein stability of SARS-CoV-2 RBD and ACE2 by the effects of mutations. The SARS-CoV-2 S structure in the open state (PDB ID: 6VYB)^35^ was collected for the comparison studies. Mutated amino acids of RBD mutants were directly replaced in the wild-type model of SARS-CoV-2 S protein.

### Molecular dynamics simulation and binding free energy analysis

All molecular dynamics (MD) simulations were performed by PMEMD.CUDA^38,39^ from AMBER 18 suite of programs^40^ on NVIDIA Geforce GTX-1070 Ti for speeding up the simulation times. Each complex structure under periodic boundary conditions was solvated in a cubic box of TIP3P water molecules extending to 10 Å along each direction from the complex model, and Na^+^ ions were added as neutralizing counterions. The cutoff distance was kept to 12 Å in order to compute the non-bonded interactions. The AMBER ff14SB force field parameters were used to apply the description of the complex characterization. The long-range electrostatic were treated using the particle mesh Ewald (PME) method^41^. The SHAKE algorithm and Langevin dynamics were applied to constrain the bonds that involved hydrogen atoms and to control the temperature. The time step of 2 fs was set and the trajectory was recorded every 0.2 ps. The temperature was gradually increased from 0 to 310.15 K over a period of 100 ps of NVT dynamics, and followed by 10 ns of NPT equilibration at 310.15 K and 1 atm pressure. Finally, a total 50 ns of the production phase MD simulations was performed for properties collection.

Amber molecular mechanics Poisson–Boltzmann surface area (MM-PBSA) approach was used to calculate the binding free energy of RBD to ACE2 using the snapshots extracted from MD simulation trajectories. The 250 numbers of structural frequencies were used to extract the structures from last 50 ns trajectories data. The 1000 snapshots were collected from the trajectory data to calculate the binding free energy by the MM-PBSA method.

All protein structure systems were used to apply the reliability of protein stability and affinity predictions. The binding free energy change was calculated using:

1 $$\Delta \Delta {\text{G}}_{decomp} = \Delta {\text{G}}_{mutation} {-} \Delta {\text{G}}_{{wild{ - }type}} .$$

A negative ΔΔG value suggests that the mutation strengthens the binding affinity and can stabilize the protein, while a positive value indicates that the mutation make protein unfavorable the RBD-ACE2 interaction.

## Supplementary Information

Supplementary Information.
