# Supplementary material for: Mutational scanning of spike RBD protein for enhanced ACE2 affinity emerging Southeast Asia in the late transmission phase
Source: Sci Rep. 2022 Apr 7;12:5896. doi: 10.1038/s41598-022-09999-9 (PMC8989122; doi:10.1038/s41598-022-09999-9)
Supplement: Supplementary file 1 — Supplementary Information. [file 41598_2022_9999_MOESM1_ESM.docx]

Supplementary materials

**Mutational scanning of spike RBD protein for enhanced ACE2 affinity emerging Southeast Asia in the late transmission phase**

Kanchanok Kodchakorn, Tawan Chokepaichitkool, Prachya Kongtawelert*

Thailand Excellence Center for Tissue Engineering and Stem Cells, Department of Biochemistry, Faculty of Medicine, Chiang Mai University, Chiang Mai 50200 Thailand

**Table of Contents**

Figure S1 Pag. 2

Figure S2 Pag. 5

Figure S3 Pag. 6

Figure S4 Pag. 7

Figure S5 Pag. 8

Figure S6 Pag. 9

Table S1 Pag. 10


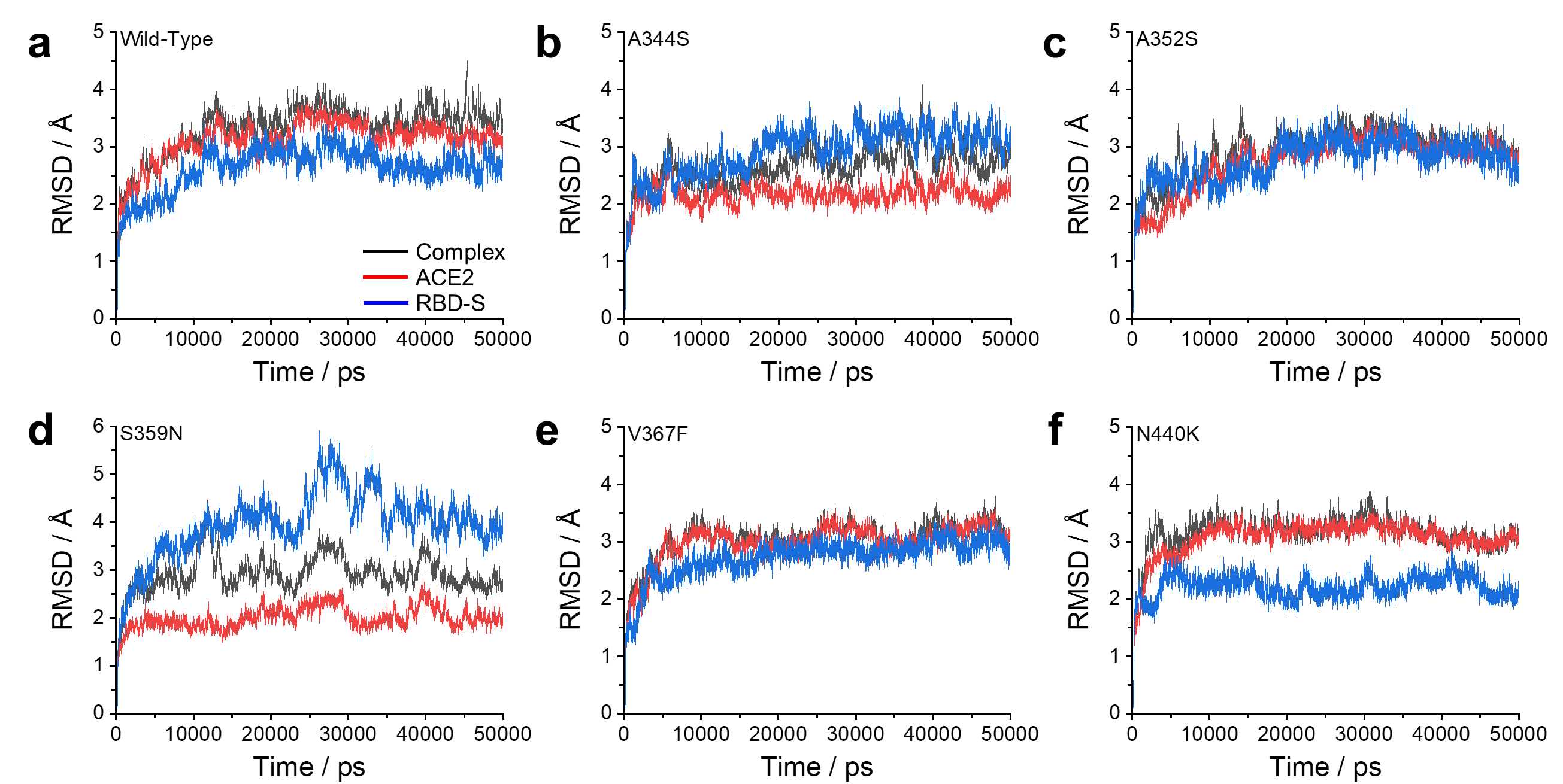


(Figure S1, *cont*.)


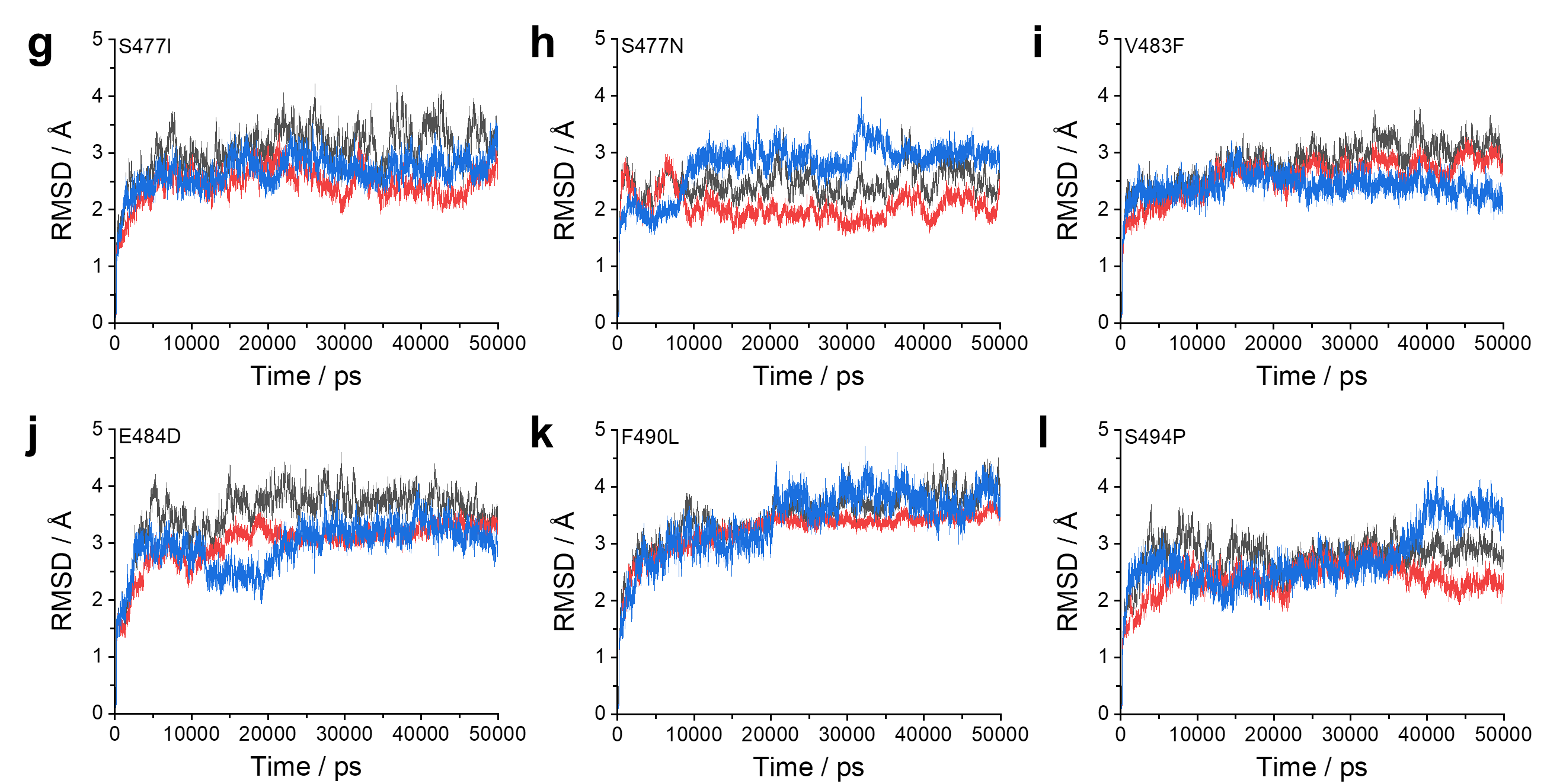


(Figure S1, *cont*.)


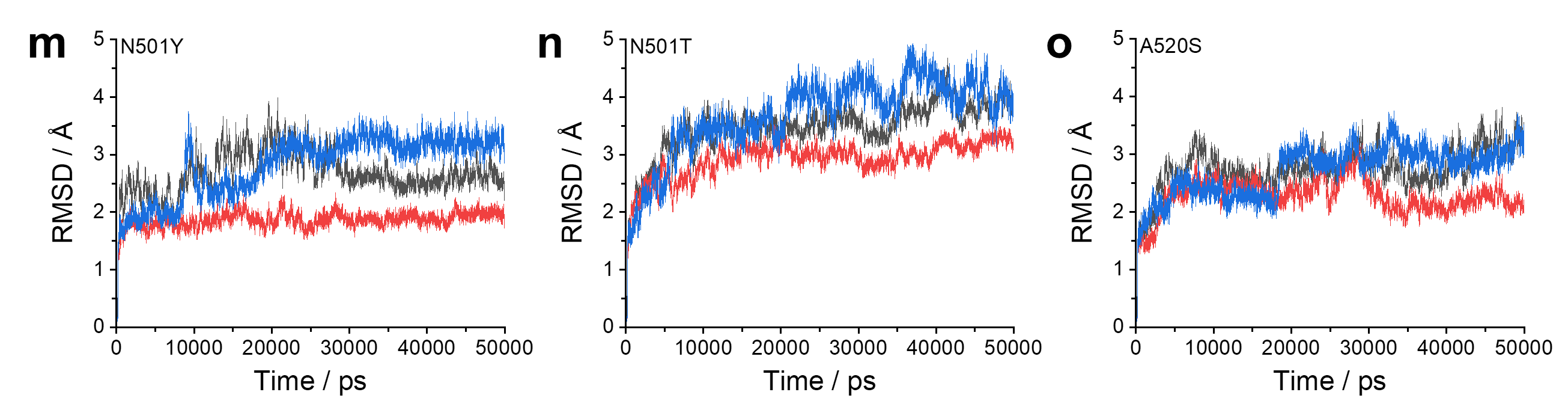


**Figure S1.** The root-mean-square deviation (RMSD) of the Cα backbone atoms of: complex (black line), ACE2 (red line), and RBD-S (blue line) protein, in each mutant system in comparison to the wild-type model.


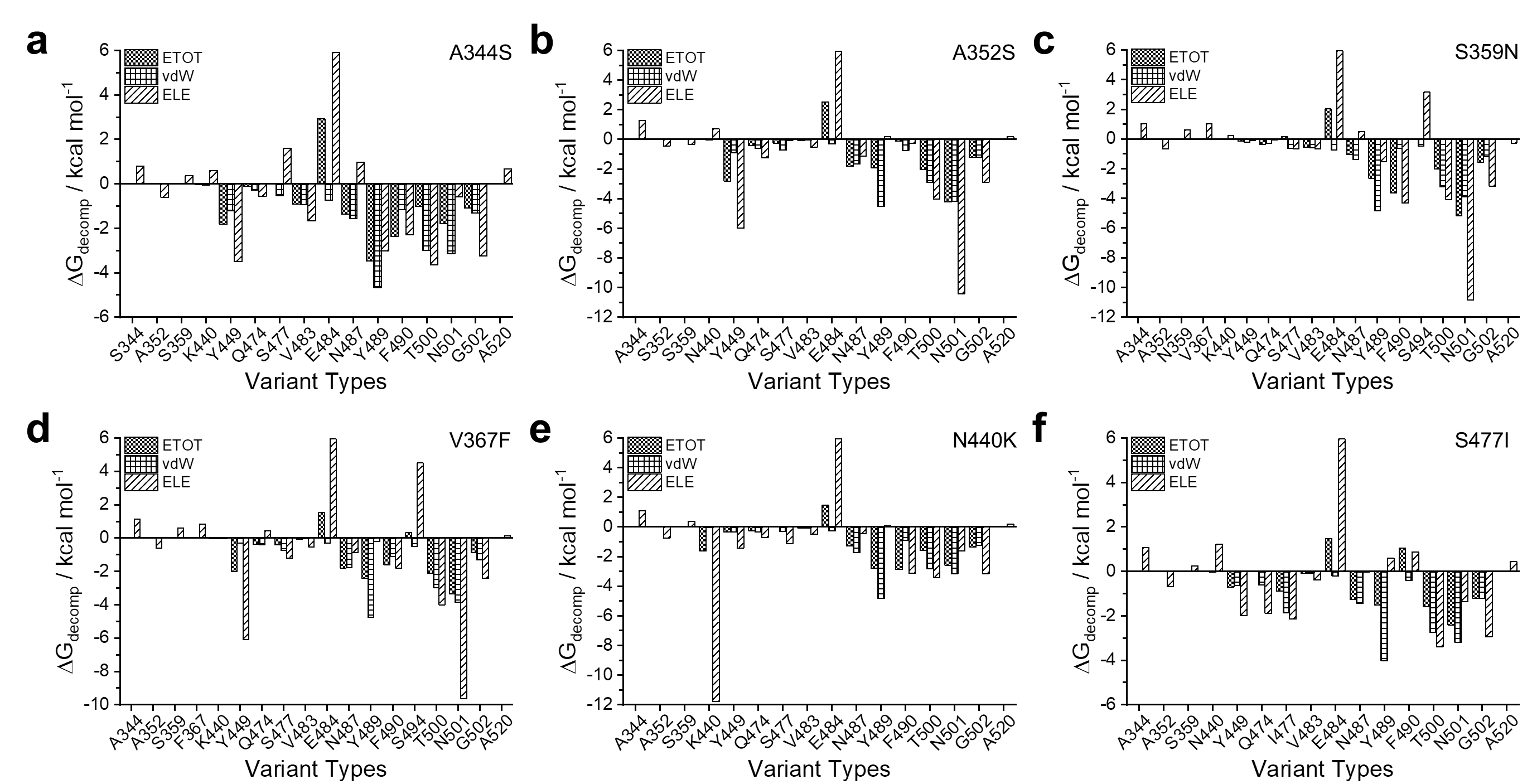


**Figure S2.** List of the decomposed binding free energy of the important residues that contributes to the RBD binding interfaces; (**a**) A344S, (**b**) A352S, (**c**) S359N, (**d**) V367F, (**e**) N440K, and (**f**) S477I, of SARS-CoV-2 RBD mutants.


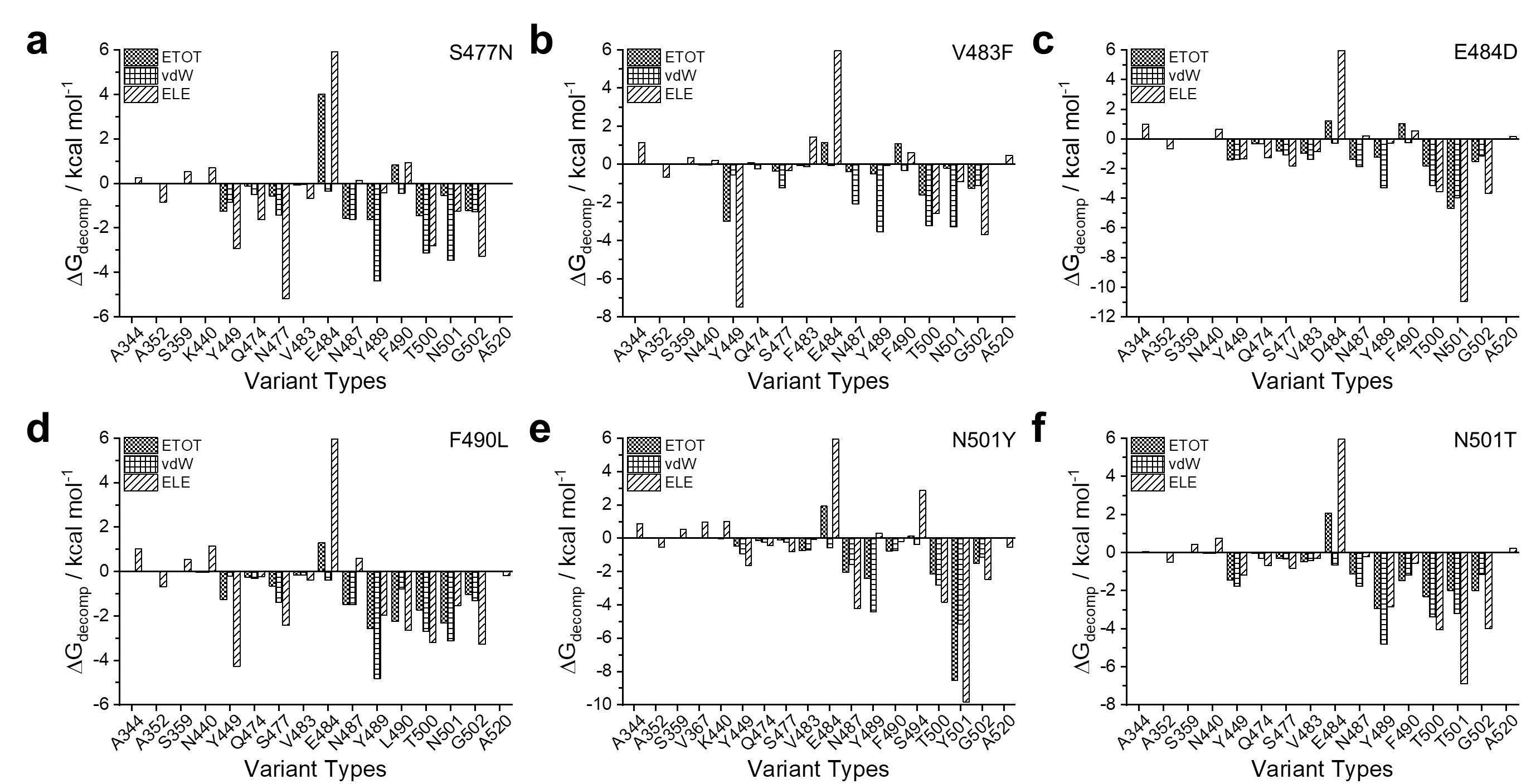


**Figure S3.** List of the decomposed binding free energy of the important residues that contributes to the RBD binding interfaces; (**a**) S477N, (**b**) V483F, (**c**) E484D, (**d**) F490L, (**e**) N501Y, and (**f**) N501T, of SARS-CoV-2 RBD mutants.


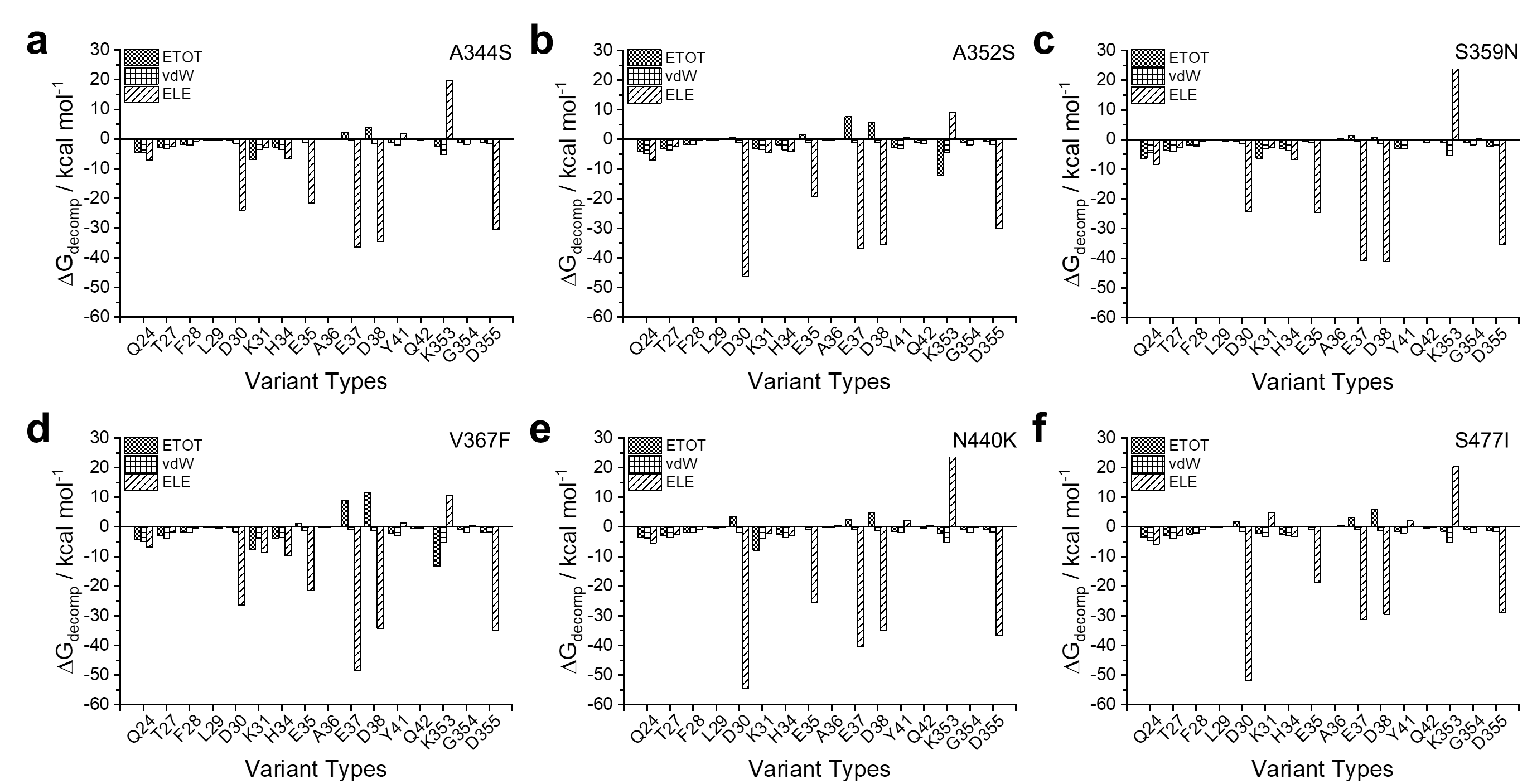


**Figure S4.** List of the decomposed binding free energy of the important residues that contributes to the ACE2 binding interfaces for; (**a**) A344S, (**b**) A352S, (**c**) S359N, (**d**) V367F, (**e**) N440K, and (**f**) S477I, of SARS-CoV-2 RBD complexes.


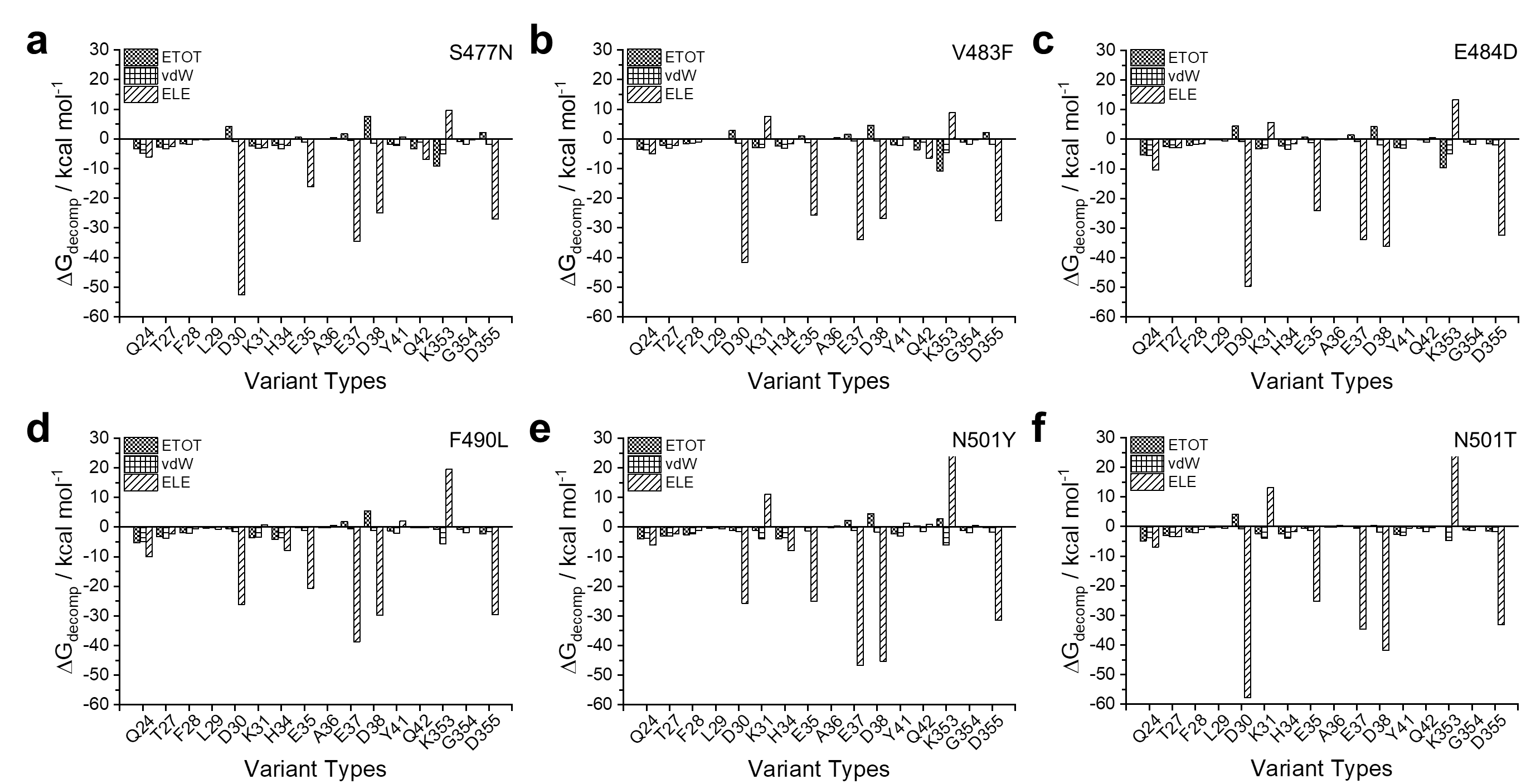


**Figure S5.** List of the decomposed binding free energy of the important residues that contributes to the ACE2 binding interfaces for; (**a**) S477N, (**b**) V483F, (**c**) E484D, (**d**) F490L, (**e**) N501Y, and (**f**) N501T, of SARS-CoV-2 RBD complexes.


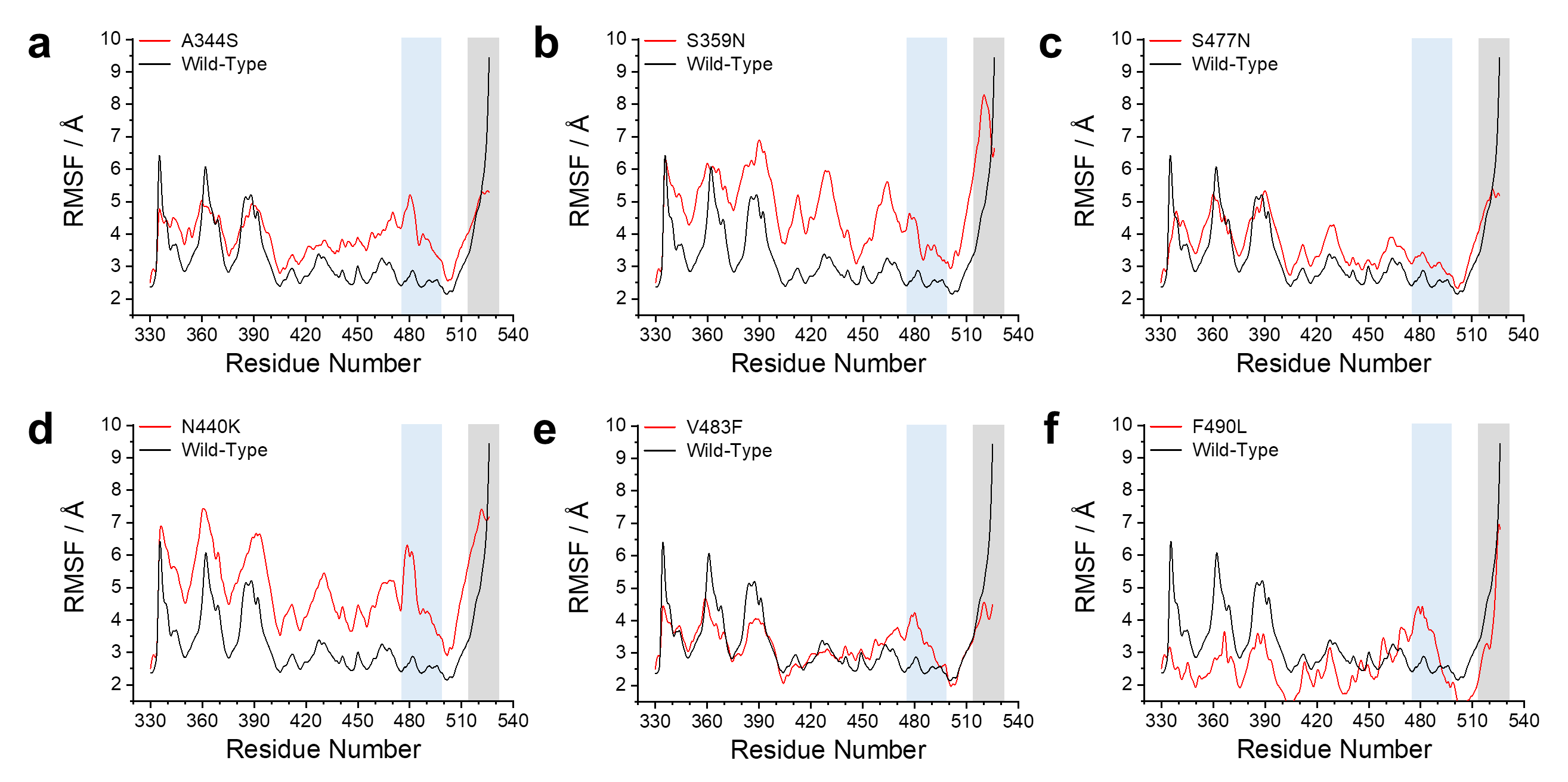


**Figure S6.** Structural dynamics of RBD mutations on the binding affinity. The root-mean-square fluctuations (RMSF) of spike RBD-ACE2 binding complex; (**a**) A344S, (**b**) S359N, (**c**) S477N, (**d**) N440K, (**e**) V483F, and (**f**) F490L, in comparison to the wild-type model. The RBD interface residues are highlighted in cyan (residues 475-500) and gray (residues 515-525) colors.

**Table S1.** Binding interface residues of SARS-CoV-2 RBD mutant with ACE2 by MD simulation.

| **Mutants** | **Amino acid position** | | **Dist_avg_ / Å** | **Bond Type** | **Secondary**  **(RBD/ACE2)** | **Molecular overlay / Å** |
| --- | --- | --- | --- | --- | --- | --- |
|  | **RBD** | **ACE2** |  |  |  |  |
| Wild-type | R403  K417  Y453  F486  F486  Y489  Q493  Q498  T500  N501  G502 | E37  D30  H34  Q24  Y83  Q24  K31  K353  D355  Y41  K353 | 5.530  1.972  2.223  1.945  1.808  2.077  2.019  2.061  1.636  2.552  1.916 | ELE  HB/ELE  HB  HB  HB  HB  HB  HB  HB  HB  HB | Sheet/Helix  Helix/Helix  Sheet/Helix  Turn/Helix  Turn/Coil  Sheet/Helix  Sheet/Helix  Coil/Turn  Turn/Coil  Coil/Helix  Coil/Turn | – |
| A344S | R403  G446  Y449  A475  G485  F486  Y489  L492  Q493  G496  T500  G502 | E37  K353  D38  S19  Y83  Q24  Y83  K31  E35  K353  D355  K353 | 4.575  2.033  1.831  1.879  1.804  1.758  3.003  2.909  1.938  2.069  2.205  1.851 | ELE  HB  HB  HB  HB  HB  HB  HB  HB  HB  HB  HB | Sheet/Turn  Coil/Turn  Turn/Helix  Coil/Coil  Turn/Coil  Coil/Helix  Sheet/Coil  Sheet/Helix  Sheet/Helix  Coil/Turn  Turn/Coil  Coil/Turn | 0.6907 |
| A352S | R403  K417  Y449  G476  F486  Y489  Q493  Q493  G496  Q498  T500  N501  G502 | E37  D30  D38  S19  Q24  Y83  K31  E35  K353  D38  Y41  K353  K353 | 2.457  1.821  1.531  2.044  2.197  1.942  1.773  1.802  2.385  1.884  1.912  2.827  1.924 | HB/ELE  HB/ELE  HB  HB  A  HB  HB  HB  HB  HB  HB  HB  HB | Sheet/Turn  Helix/Helix  Coil/Helix  Coil/Coil  Coil/Helix  Sheet/Coil  Sheet/Helix  Sheet/Turn  Coil/Turn  Coil/Helix  Turn/Helix  Coil/Turn  Coil/Turn | 0.6718 |
| S359N | R403  G446  G485  F486  Y489  F490  Q493  Q493  Q498  T500  G502 | E37  Q42  Y83  Q24  Q24  K31  K31  E35  K353  D355  K353 | 4.686  1.772  2.016  2.014  1.997  1.825  1.797  1.971  1.724  1.675  1.770 | ELE  HB  HB  HB  HB  HB  HB  HB  HB  HB  HB | Sheet/Helix  Turn/Helix  Turn/Coil  Turn/Helix  Sheet/Helix  Coil/Helix  Sheet/Helix  Sheet/Helix  Coil/Turn  Turn/Coil  Coil/Turn | 0.6655 |
| V367F | R403  K417  K417  E484  F486  Q493  Y495  Q498  Q498  T500  N501  G502 | E37  D30  H34  K31  Q24  H34  K353  K353  D38  D355  K353  K353 | 3.060  4.818  2.356  5.161  1.979  2.344  1.939  1.928  1.890  1.563  2.391  2.276 | HB/ELE  ELE  HB  ELE  HB  HB  HB  HB  HB  HB  HB  HB | Sheet/Turn  Helix/Helix  Helix/Turn  Coil/Helix  Turn/Helix  Sheet/Turn  Coil/Turn  Coil/Turn  Coil/Helix  Turn/Coil  Coil/Turn  Coil/Turn | 0.7282 |
| N440K | R403  K417  E484  F486  Y489  F490  Q493  Q493  G496  T500  G502 | E37  D30  K31  Q24  Y83  K31  K31  E35  K353  D355  K353 | 5.299  2.761  5.294  1.758  3.079  1.793  2.013  1.749  2.711  1.780  1.978 | ELE  ELE  ELE  HB  HB  HB  HB  HB  HB  HB  HB | Sheet/Helix  Helix/Helix  Coil/Helix  Coil/Helix  Sheet/Coil  Coil/Helix  Sheet/Helix  Sheet/Turn  Coil/Turn  Turn/Coil  Coil/Turn | 0.7003 |
| S477I | R403  K417  Y453  F486  N487  Q493  Q493  G496  T500  G502 | E37  D30  H34  Q24  Q24  K31  E35  K353  D355  K353 | 5.062  2.038  2.186  1.913  2.830  1.958  1.796  2.020  1.591  1.853 | ELE  HB/ELE  HB  HB  HB  HB  HB  HB  HB  HB | Sheet/Helix  Helix/Helix  Sheet/Helix  Coil/Helix  Coil/Helix  Sheet/Helix  Sheet/Helix  Coil/Turn  Turn/Coil  Coil/Turn | 0.6489 |
| S477N | R403  K417  G446  Y449  E484  F486  Q493  Q493  G496  Q498  T500  G502 | E37  D30  Q42  D38  K31  Y83  E35  H34  K353  Q42  N330  K353 | 4.691  1.796  2.764  1.590  3.110  2.677  1.803  2.105  2.240  1.856  2.061  2.471 | ELE  HB/ELE  HB  HB  HB/ELE  HB  HB  HB  HB  HB  HB  HB | Sheet/Turn  Helix/Helix  Turn/Helix  Coil/Helix  Coil/Helix  Coil/Coil  Sheet/Helix  Sheet/Helix  Coil/Turn  Coil/Helix  Turn/Helix  Coil/Turn | 0.7305 |
| V483F | K417  Y449  A475  S477  N487  L492  Q493  Q493  G496  Q498  Q498  T500  G502 | D30  D38  Q24  Q24  Y83  K31  K31  E35  K353  Q42  K353  D355  K353 | 1.950  2.001  1.949  2.195  1.949  3.059  1.733  2.115  2.358  2.106  1.783  2.303  1.869 | HB/ELE  HB  HB  HB  HB  HB  HB  HB  HB  HB  HB  HB  HB | Helix/Helix  Coil/Helix  Coil/Helix  Coil/Helix  Coil/Coil  Sheet/Helix  Sheet/Helix  Sheet/Helix  Coil/Turn  Coil/Helix  Coil/Turn  Turn/Coil  Coil/Turn | 0.7228 |
| E484D | R403  K417  Y449  F486  Q493  Q493  G496  Q498  T500  G502 | E37  D30  Q42  Q24  K31  E35  K353  D38  D355  K353 | 5.028  1.950  2.423  1.997  2.010  2.018  1.834  1.805  1.829  2.098 | ELE  HB/ELE  HB  HB  HB  HB  HB  HB  HB  HB | Sheet/Helix  Helix/Helix  Coil/Helix  Helix/Helix  Sheet/Helix  Sheet/Helix  Coil/Turn  Coil/Helix  Turn/Coil  Coil/Turn | 0.7311 |
| F490L | R403  F486  F486  Y489  L490  Q493  Q493  G496  T500  G502  Y505 | E37  Q24  Y83  Q24  K31  K31  E35  K353  D355  K353  A386 | 4.960  1.971  1.833  2.918  2.102  1.723  1.901  1.729  1.663  2.288  1.964 | ELE  HB  HB  HB  HB  HB  HB  HB  HB  HB  HB | Sheet/Helix  Coil/Helix  Coil/Coil  Sheet/Helix  Coil/Helix  Sheet/Helix  Sheet/Helix  Coil/Turn  Turn/Coil  Coil/Turn  Turn/Turn | 0.6347 |
| S494P | R403  K417  N487  N487  Q493  G496  T500  G502 | E37  D30  Y83  Q24  E35  K353  D355  K353 | 5.424  1.868  2.109  2.905  1.709  2.071  1.756  1.700 | ELE  HB/ELE  HB  HB  HB  HB  HB  HB | Sheet/Turn  Turn/Helix  Coil/Coil  Coil/Helix  Sheet/Helix  Coil/Turn  Turn/Coil  Coil/Turn | 0.7054 |
| N501Y | R403  R403  F486  N487  Q493  T500  Y501  Y501  G502  Y505 | E37  E37  Y83  Q24  E35  D355  K353  D38  K353  A386 | 1.969  4.942  2.448  1.868  1.881  1.833  4.649  1.780  1.790  1.903 | HB/ELE  ELE  HB  HB  HB  HB  A  HB  HB  HB | Sheet/Helix  Sheet/Helix  Turn/Coil  Turn/Helix  Sheet/Helix  Turn/Coil  Coil/Turn  Coil/Helix  Coil/Turn  Turn/Turn | 0.6778 |
| N501T | K417  F486  F486  L492  Q493  Q493  Q498  T500  T501  G502 | D30  Q24  Y83  K31  K31  E35  D38  Y41  K353  K353 | 1.714  2.335  2.077  2.140  1.805  1.887  1.694  1.665  2.277  1.955 | HB/ELE  HB  HB  HB  HB  HB  HB  HB  HB  HB | Helix/Helix  Turn/Helix  Turn/Coil  Sheet/Helix  Sheet/Helix  Sheet/Helix  Coil/Helix  Turn/Helix  Coil/Turn  Coil/Turn | 0.7412 |
| A520S | K417  E484  F486  Y489  Q493  S494  G496  Q498  T500  G502  Y505 | D30  K31  Y83  Q24  E35  H34  K353  Y41  D355  K353  A386 | 1.902  1.816  1.787  2.448  2.269  1.952  1.740  2.775  1.831  2.019  1.733 | HB/ELE  HB/ELE  HB  HB  HB  HB  HB  HB  HB  HB  HB | Helix/Helix  Coil/Helix  Turn/Coil  Coil/Helix  Sheet/Turn  Sheet/Turn  Coil/Turn  Coil/Helix  Turn/Coil  Coil/Turn  Helix/Turn | 0.6563 |

HB, hydrogen bond; A, Pi-donor hydrogen bond; ELE, electrostatic.

Dist_avg_ (Å) = Average distance between acceptor and donor hydrogen bond.

Molecular overlay = Aligns the selected molecule to wild-type molecule. The similarity coefficients can assume values by the ratio of the selected molecule and target molecule.
